# Comparison of biomarker based Matrix Assisted Laser Desorption Ionization-Time of Flight Mass Spectrometry (MALDI-TOF MS) and conventional methods in the identification of clinically relevant bacteria and yeast

**DOI:** 10.1186/s12866-017-1037-z

**Published:** 2017-05-25

**Authors:** Ali Kassim, Valentin Pflüger, Zul Premji, Claudia Daubenberger, Gunturu Revathi

**Affiliations:** 1 Aga Khan University Hospital, Nairobi, Kenya; 2 Swiss Tropical and Public Health Institute, Basel, Switzerland; 3 Mabritec AG, Riehen, Switzerland

**Keywords:** MALDI-TOF MS, PAPMID™, VITEK 2™, SARAMIS™

## Abstract

**Background:**

MALDI-TOF MS is an analytical method that has recently become integral in the identification of microorganisms in clinical laboratories. It relies on databases that majorly employ pattern recognition or fingerprinting. Biomarker based databases have also been developed and there is optimism that these may be superior to pattern recognition based databases. This study compared the performance of ribosomal biomarker based MALDI-TOF MS and conventional methods in the identification of selected bacteria and yeast.

**Methods:**

The study was a cross sectional study identifying clinically relevant bacteria and yeast isolated from varied clinical specimens submitted to a clinical laboratory. The identification of bacteria using conventional Vitek 2™ automated system, serotyping and MALDI-TOF MS was performed as per standard operating procedures. Comparison of sensitivities were then carried out using Pearson Chi-Square test and *p*-value of <0.05 was considered statistically significant. Secondary outcomes analyzed included the major and minor error rates.

**Results:**

Of the 383 isolates MALDI-TOF MS and conventional methods identified 97.6 and 95.7% (*p* = 0.231) to the genus level and 97.4 and 88.0% (*p* = 0.000) to the species level respectively. Biomarker based MALDI-TOF MS was significantly superior to Vitek 2™ in the identification of Gram negative bacteria and Gram positive bacteria to the species level. For the Gram positive bacteria, significant difference was observed in the identification of Coagulase negative *Staphylococci* (*p* = 0.000) and *Enterococcus* (*p* = 0.008). Significant difference was also observed between serotyping and MALDI-TOF MS (*p* = 0.005) and this was attributed to the lack of identification of *Shigella* species by MALDI-TOF MS. There was no significant difference observed in the identification of yeast however some species of *Candida* were unidentified by MALDI-TOF MS.

**Conclusion:**

Biomarker based MALDI-TOF MS had good performance in a clinical laboratory setting with high sensitivities in the identification of clinically relevant microorganisms.

## Background

Matrix Assisted Laser Desorption Ionization-Time of Flight Mass Spectrometry (MALDI-TOF MS) is an analytical method developed in mid-1980s that has evolved rapidly to fingerprint spectra for various microorganisms including bacteria and fungi by analyzing protein profiles [1–5]. Databases were subsequently developed and adopted in clinical microbiology laboratories for the identification of clinically relevant microorganisms. These databases employ the concept of pattern recognition or fingerprinting where mass spectra obtained from a bacteria or yeast is compared to the existing spectra in the databases to find the closest match [6, 7].

The biomarker approach to identification of bacteria uses the specific proteins found within the bacterial cells. Ribosomal proteins have turned out to be one of the ideal biomarkers because they are abundant, highly conserved and encoded by chromosomal genes. They also have molecular masses that fall within the 4 to 30 kDa range of MALDI-TOF MS [8]. Despite being highly conserved there are inter-species and inter-strain differences that can be employed in typing and sub-typing of micro-organisms. Using ribosomal biomarkers Suarez et al. were able to group various strains of *Neisseria meningitidis* into six subgroups that corresponded to sequence types and/or clonal complexes [9].

The Putative Assigned Protein Masses for Identification Database (PAPMID™) (Mabritec AG, Switzerland) is a biomarker based database that comprises molecular masses of ribosomal proteins calculated from partial or whole bacterial genome sequences. This database has been shown to supplement pattern recognition reference databases like the SARAMIS™ database [8]. Ziegler et al. found that it performed as well as 16S rRNA sequencing in the correct identification of root nodule bacteria [8]. This approach has also been shown to differentiate strains of *Acinetobacter* Genomic species 13BJ/14TU that are intrinsically resistant to polymixins from *Acinetobacter haemolyticus* [10]. This database therefore has the ability to be an easily accessible and affordable alternative to gene sequencing especially for resource poor settings in the developing world. We set out to compare the sensitivity of biomarker based MALDI-TOF MS to conventional methods like Vitek 2 and serotyping in the identification of bacteria and yeast from a clinical microbiology laboratory.

## Methods

The study is a cross sectional study carried out at the Aga Khan University Hospital, Nairobi, Kenya (AKUH, N) and Mabritec laboratory, Riehen, Switzerland. Ethical approval was granted by the AKUH, N’s Research and Ethics Committee (Ref 2016/REC-06). Clinically relevant bacteria and yeasts identified from clinical specimens submitted to the AKUH, N laboratories were included in the study. The specimens were given special codes and delinked from patient identifiers throughout the study. Only specimens classified as ‘UN3373 Biological Substances Cat B’ were shipped under ‘Dry Ice UN 1845’ for the MALDI TOF analysis at Mabritec AG. Blinding was maintained throughout the various stages of the study.

### Processing of samples using Vitek 2™ and serotyping

The processing of the clinical specimen and identification of bacteria using conventional Vitek 2™ automated system was done at AKUH, N. Standard operating procedures (SOPs) in processing and culture of these specimens were strictly adhered to and the organisms were then put through the Vitek 2™ automated system for the final biochemical identification. Serotyping was employed for the identification of some isolates including *Streptococcus*, *Salmonella* and *Shigella* species.

### Processing of samples for MALDI TOF MS analyses

Freshly cultured isolates were spotted in duplicates directly onto MALDI TOF target plates. The spots were then overlaid with 1 ul of 25% formic acid and allowed to air dry. They were then overlaid with 1 ul of matrix solution consisting of 40 g of Alpha–cyano-4-hydroxycinnamic acid (CHCA; Sigma-Aldrich, Buchs, Switzerland) in 33% ethanol, 33% deionized water, 33% acetonitrile (ACN) (Sigma-Aldrich) and 3% trifluoroacetic acid (TFA). For the preparation of yeast, a formic acid suspension protocol was used instead of direct smear. A colony of yeast was picked using a 1 ul plastic inoculation loop and suspended in 20 ul of 25% formic acid. One microliter of this suspension was then spotted onto the MALDI plate, allowed to dry and then overlaid with the matrix. The matrix was then allowed to dry in room air.

The MALDI plates were loaded onto the Axima™ Confidence (Shimadzu-Biotech Corp., Kyoto, Japan) mass spectrometer and mass spectra obtained in positive linear mode at a frequency of 50 Hz and within mass range of 3000 Da to 20,000 Da. Each MALDI plate was externally calibrated using a spectra of reference strain of *Escherichia coli* DH5α (Invitrogen, Carlsbad, USA) that was also spotted onto the plates.

### Data acquisition and analysis using SARAMIS™ and PAPMID™

Empiric spectra for each spot was acquired and an average of 50 to 100 protein mass fingerprints were processed using the Launchpad™ 2.8 software (Shimadzu-Biotech). The spectra were then analyzed using the Saramis™ database and matched with the SuperSpectra™ to look for the closest match. The spectra was then compared to the PAPMID™ database to look for matches in species or strain specific ribosomal biomarkers. The closest match was taken to be the identification of the microorganism.

### Data analysis

Data collected were entered into Excel worksheets and analyzed using SPSS version 23.0 (IBM; Armonk, New York, USA). Comparison of sensitivities were then carried out using Pearson Chi-Square test and *P*-value of <0.05 was considered statistically significant. Secondary outcomes analyzed include the major and minor error rates reported in percentages.

## Results

The 383 isolates recruited included 222 Gram negative bacteria, 131 Gram positive bacteria and 30 yeast. Of all the isolates, biomarker based MALDI-TOF MS identified 97.6% correctly to the genus level while the conventional methods identified 95.7% to the genus level with a *p*-value of 0.231. At the species level, 358 isolates were analyzed. Of these, MALDI-TOF MS identified 97.4% correctly while conventional methods identified only 88.0% correctly with a significant *p*-value of 0.000.

In Table 1 below the sensitivities of 195 Gram negative bacteria identified using Vitek 2 and biomarker based MALDI-TOF MS are shown. Of these, 100 and 92.3% (*p* = 0.000) were correctly identified to the genus level while 100 and 88.2% (*p* = 0.000) were correctly identified to the species level by MALDI-TOF MS and Vitek 2™ respectively. Vitek 2 correctly identified 48 out of 55 *E. coli* isolates to both the genus and species levels while MALDI TOF MS identified all correctly with a significant *p*-value of 0.006. Vitek 2™ misidentified four isolates of *E. coli* as *Serratia liquefaciens* and *Serratia fonticola*. The other three isolates of *E. coli* were misidentified as *Klebsiella pneumoniae*, *Pseudomonas aeruginosa* and *Moraxella* species. One isolate of *E. coli* was identified correctly by both PAPMID™ database and Vitek 2™ while SARAMIS™ database misidentified it as *Shigella sonnei*.

**Table 1:** Comparison of the sensitivities of Vitek 2 and MALDI-TOF MS in the identification of Gram negative bacteria to the species and genus levels

Organism (*n*)	Number (%) of isolates with Correct identification to the species level:			Number (%) of isolates with Correct identification to the genus level:		
	Vitek 2	MALDI-TOF MS	*P* value	Vitek 2	MALDI-TOF MS	*P* value
*E coli* (55)	48 (87.3)	55 (100.0)	0.006	48 (87.3)	55 (100.0)	0.006
*Klebsiella pneumoniae* (33)	32 (97.0)	33 (100.0)	0.314	32 (97.0)	33 (100.0)	0.314
*Klebsiella oxytoca* (4)	3 (75.0)	4 (100.0)	0.285	4 (100.0)	4 (100.0)	
*Pseudomonas aeruginosa* (14)	14 (100.0)	14 (100.0)		14 (100.0)	14 (100.0)	
*Pseudomonas mendocina* (1)	0 (0.0)	1 (100.0)		1 (100.0)	1 (100.0)	
*Pseudomonas pseudoalcaligenes* (1)	0 (0.0)	1 (100.0)		1 (100.0)	1 (100.0)	
*Acinetobacter baumannii* (18)	18 (100.0)	18 (100.0)		18 (100.0)	18 (100.0)	
*Acinetobacter genomospecies* 13BJ/14TU (1)	0 (0.0)	1 (100.0)		1 (100.0)	1 (100.0)	
*Acinetobacter ursingii* (1)	1 (100.0)	1 (100.0)		1 (100.0)	1 (100.0)	
*Citrobacter freundii* (6)	5 (83.3)	6 (100.0)	0.296	6 (100.0)	6 (100.0)	
*Citrobacter koseri* (3)	3 (100.0)	3 (100.0)		3 (100.0)	3 (100.0)	
*Enterobacter cloacae* (12)	8 (66.7)	12 (100.0)	0.028	9 (75.0)	12 (100.0)	0.064
*Enterobacter aerogenes* (5)	5 (100.0)	5 (100.0)		5 (100.0)	5 (100.0)	
*Enterobacter gergoviae* (1)	1 (100.0)	1 (100.0)		1 (100.0)	1 (100.0)	
*Haemophilus influenzae* (4)	3 (75.0)	4 (100.0)	0.285	3 (75.0)	4 (100.0)	
*Morganella morganii* (8)	7 (87.5)	8 (100.0)	0.302	8 (100.0)	8 (100.0)	
*Proteus mirabilis* (7)	7 (100.0)	7 (100.0)		7 (100.0)	7 (100.0)	
*Proteus penneri/vulgaris* (7)	6 (85.7)	7 (100.0)	0.299	7 (100.0)	7 (100.0)	
*Providencia rettgeri* (1)	1 (100.0)	1 (100.0)		1 (100.0)	1 (100.0)	
*Stenotrophomonas maltophilia* (10)	7 (70.0)	10 (100.0)	0.06	7 (70.0)	10 (100.0)	0.06
*Serratia marcescens* (1)	1 (100.0)	1 (100.0)		1 (100.0)	1 (100.0)	
*Vibrio alginolyticus* (1)	1 (100.0)	1 (100.0)		1 (100.0)	1 (100.0)	
*Elizabethkingia meningoseptica* (1)	1 (100.0)	1 (100.0)		1 (100.0)	1 (100.0)	
Total (195)	172 (88.2)	195 (100.0)	0.000	180 (92.3)	195 (100.0)	0.000

Of the Gram positive bacteria, 111 were identified routinely using Vitek 2™ while the rest were routinely identified using serotyping. Table 2 below shows the sensitivities of Gram positive bacteria identified using Vitek 2™ and MALDI-TOF MS. Of these, 100% were correctly identified to both the genus and species levels by MALDI-TOF MS while 99.1 and 83.8% were correctly identified to the genus and species levels respectively by Vitek 2™ with a significant *P* value of 0.000. All *Staphylococcus* species were identified correctly to the genus level by both Vitek 2™ and MALDI-TOF MS. However, Vitek 2™ correctly identified only 77.1% of Coagulase negative *Staphylococcus* to the species level while MALDI-TOF MS identified all correctly. All *Enterococcus* species were correctly identified to the species level by MALDI-TOF MS while Vitek 2™ identified only 72.7% correctly to the species level.

**Table 2:** Comparison of the sensitivities of Vitek 2 and MALDI-TOF MS in the identification of Gram positive bacteria to the species and genus levels

Organism (*n*)	Number (%) of isolates with Correct identification to the species level:			Number (%) of isolates with Correct identification to the genus level:		
	Vitek 2	MALDI-TOF MS	*P* value	Vitek 2	MALDI-TOF MS	*P* value
*Staph aureus* (33)	33 (100.0)	33 (100.0)		33 (100.0)	33 (100.0)	
CoNS (48)	37 (77.1)	48 (100.0)	0.000	48 (100.0)	48 (100.0)	
*Staph capitis* (3)	3 (100.0)	3 (100.0)		3 (100.0)	3 (100.0)	
*Staph cohnii* (1)	1 (100.0)	1 (100.0)		1 (100.0)	1 (100.0)	
*Staph epidermidis* (10)	9 (90.0)	10 (100.0)	0.305	10 (100.0)	10 (100.0)	
*Staph hemolyticus* (6)	3 (50.0)	6 (100.0)	0.046	6 (100.0)	6 (100.0)	
*Staph hominis* (2)	2 (100.0)	2 (100.0)		2 (100.0)	2 (100.0)	
*Staph saprophyticus* (23)	18 (78.3)	23 (100.0)	0.018	23 (100.0)	23 (100.0)	
*Staph sciuri* (1)	1 (100.0)	1 (100.0)		1 (100.0)	1 (100.0)	
*Staph simulans* (1)	0 (0.0)	1 (100.0)		1 (100.0)	1 (100.0)	
*Staph succinus* (1)	0 (0.0)	1 (100.0)		1 (100.0)	1 (100.0)	
*Strep mitis/oralis* (4)	3 (75.0)	4 (100.0)	0.285	4 (100.0)	4 (100.0)	
*Strep pneumonia*e (3)	3 (100.0)	3 (100.0)		3 (100.0)	3 (100.0)	
*Enterococcus* (22)	16 (72.7)	22 (100.0)	0.008	21 (95.5)	22 (100.0)	
*Enterococcus avium* (1)	1 (100.0)	1 (100.0)		1 (100.0)	1 (100.0)	
*Enterococcus faecalis* (11)	9 (81.8)	11 (100.0)	0.138	10 (90.9)	11 (100.0)	0.306
*Enterococcus faecium* (7)	4 (57.1)	7 (100.0)	0.051	7 (100.0)	7 (100.0)	
*Enterococcus gallinarum* (1)	1 (100.0)	1 (100.0)		1 (100.0)	1 (100.0)	
*Enterococcus hirae* (2)	1 (50.0)	2 (100.0)	0.248	2 (100.0)	2 (100.0)	
*Aerococcus viridans* (1)	1 (100.0)	1 (100.0)		1 (100.0)	1 (100.0)	
Total (111)	93 (83.8)	111 (100.0)	0.000	110 (99.1)	111 (100.0)	0.316

All the 18 *Salmonella* isolates were correctly identified by both MALDI-TOF MS and serotyping to the genus level. However, no comparison was performed at the species level. All 9 *Shigella* isolates were misidentified as *E. coli* by MALDI-TOF MS. All 20 Gram positive bacteria, mainly *Streptococcus* species, were identified correctly to the genus level by both MALDI-TOF MS and Serotyping while only one was misidentified to the species level by serotyping. Table 3 below shows the sensitivities of serotyping and MALDI-TOF MS in the identification of some Gram negative and Gram positive bacteria routinely identified using serotyping.

**Table 3:** Comparison of the sensitivities of serotyping and MALDI-TOF MS in the identification of selected bacteria to the species and genus levels

Organism (*n*)	Number (%) of isolates with Correct identification to species level:			Number (%) of isolates with Correct identification to genus level:		
	Serotyping	MALDI-TOF MS	*P* value	Serotyping	MALDI-TOF MS	*P* value
*Salmonella sp.* (18)	-	-		18 (100.0)	18 (100.0)	
*Shigella flexneri* (6)	6 (100.0)	0	0.001	6 (100.0)	0	0.001
*Shigella sonnei* (2)	2 (100.0)	0	0.046	2 (100.0)	0	0.046
*Shigella dysentriae* (1)	1 (100.0)	0	0.157	1 (100.0)	0	0.157
*Strep agalactiae* (13)	12 (92.3)	13 (100.0)	0.308	13 (100.0)	13 (100.0)	
*Strep pyogenes* (7)	7 (100.0)	7 (100.0)		7 (100.0)	7 (100.0)	
Total (29/47)	28 (96.6)	20 (69.0)	0.005	47 (100.0)	38 (80.9)	0.002

Table 4 below shows the comparison of sensitivities of biomarker based MALDI-TOF MS and Vitek 2™ in the identification of yeasts. Of the 30 yeast isolates, 23 were correctly identified to the genus level by both MALDI-TOF MS and Vitek 2™ while 23 and 22 were identified correctly to the species level by MALDI-TOF MS and Vitek 2™ respectively. Six isolates of *Candida haemulonii* and 1 isolate of *Candida guillermondii* that were unidentified by MALDI-TOF MS were excluded from analysis.

**Table 4:** Comparison of the sensitivities of Vitek 2 and MALDI-TOF MS in the identification of selected yeast to the species and genus levels

Organism (n)	Number (%) of isolates with Correct identification to the species level:			Number (%) of isolates with Correct identification to the genus level:		
	Vitek	MALDI-TOF MS	*P* value	Vitek	MALDI-TOF MS	*P* value
*Candida albicans* (5)	5	5		5	5	
*Candida dubliniensis* (2)	2	2		2	2	
*Candida glabrata* (4)	4	4		4	4	
*Candida guillermondii* (1)^a^	-	-		-	-	
*Candida haemulonii* (6)^a^	-	-		-	-	
*Candida kefyr* (1)	1	1		1	1	
*Candida krusei* (4)	4	4		4	4	
*Candida parapsilosis* (2)	2	2		2	2	
*Candida tropicalis* (4)	3	4	0.285	4	4	
*Cryptococcus neoformans* (1)	1	1		1	1	
Total (23)	22 (95.7)	23 (100.0)	0.312	23 (100.0)	23 (100.0)	

KEY: ^a^6 isolates of *Candida haemulonii* and 1 isolate of *Candida guillermondii* have been excluded from analysis as they were not identified by MALDI-TOF MS.

Overall, MALDI-TOF MS had 2.6% minor errors while conventional methods had 12.0% (*p* = 0.000). The major errors were noted to be at 2.4 and 4.3% (*p* = 0.231) for MALDI-TOF MS and conventional methods respectively. No errors were made by MALDI-TOF in the identification of Gram positive bacteria in comparison to 16.2% (*p* = 0.000) minor errors and 0.9% (*p* = 0.316) major errors noted for Vitek 2™. Vitek 2™ had 11.8% (*p* = 0.000) minor errors and 7.7% (*p* = 0.000) major errors in the identification of Gram negative bacteria.

## Discussion

MALDI TOF MS has been shown to reduce the turnaround time, hospital stays and costs as compared to biochemical based tests [11]. Thus far, the MALDI-TOF MS databases introduced into clinical laboratories have employed pattern recognition approaches in the identification of microorganisms. In an attempt to improve sensitivities, use of specific biomarkers rather than generic non-conserved markers in the databases led to the concept of biomarker based approach in identification [9]. The Putative Assigned Protein Masses for Identification Database (PAPMID™) (Mabritec AG, Switzerland) is a biomarker based database that comprises molecular masses of ribosomal proteins calculated from partial or whole bacterial genome sequences [8].

Our study included an assortment of bacteria and yeast isolated in a routine clinical laboratory using Vitek 2 and serotyping. Guo et al. showed an overall sensitivity of 99.6 and 93.37% in the identification of bacteria to the genus and species levels respectively [12]. The sensitivity of biomarker based MALDI-TOF MS in the identification of Gram negative bacteria to the genus and species level was 99 and 92% respectively. This was significantly better than that of the conventional methods including Vitek 2. Studies by Wang et al. show sensitivities of pattern recognition MALDI TOF MS in the identification of Gram negative bacteria ranging from 93.2 to 98.7% [13]. *Shigella* species that had been identified routinely using serotyping were all identified as *E. coli* by MALDI-TOF MS. Studies have shown the difficulty in discriminating these two species due to their close relationship [14–16]. All the *Salmonella* isolates were identified by MALDI-TOF MS as *Salmonella enterica* subsp. *enterica* except one that was identified as *Salmonella* species. No comparison was done at the species level as serotyping identified serogroups as per the Kauffmann-White Scheme while MALDI-TOF MS identified species and subspecies [17]. Serological and biochemical tests would still be recommended in confirmation of identification of *Salmonella* and *Shigella* species [18]. An isolate of *Acinetobacter* genomospecies 13BJ/14TU by PAPMID™ was identified by SARAMIS™ as *Acinetobacter* species and as *Acinetobacter baumannii* by Vitek 2™. The *Acinetobacter* genomospecies 13BJ/14TU have been shown to be intrinsically resistant to colistin hence the significance of correctly differentiating it from other *Acinetobacter* species [10, 19].

In the overall identification of Gram positive bacteria to the genus level, there was no significant difference in the sensitivities between MALDI TOF MS and conventional methods. However significant difference was observed in the identification of these bacteria to the species level. Coagulase negative *Staphylococci* (CoNS) were identified to the species level with a sensitivity of 100% by biomarker MALDI-TOF MS compared to 77.5% by Vitek 2™ (*p* = 0.000). This sensitivity was slightly better than that shown by Zhou et al. where pattern recognition MALDI-TOF MS identified 97.7% of CoNS correctly to the species level compared to 76.0% by an automated biochemical system [20]. The significance of correctly identifying CoNS especially lies in the fact that they are the commonest organisms found in positive blood cultures and the need to rule out surface contamination and reduce cost of unnecessary interventions [13, 20].

In the identification of *Enterococcus*, biomarker MALDI-TOF MS correctly identified all to the species level while Vitek 2™ identified only 72.7% correctly (*P* = 0.008). There was no clinically significant difference between the two methods in the identification of *Enterococcus* to the genus level. These findings mirror those of previous studies that show excellent identification of *Enterococcus* by MALDI-TOF MS especially in the discrimination between *E. faecalis* and *E. faecium* due to their significant differences in their resistance patterns [20, 21].

In the identification of *Streptococcus*, there was no significant difference between biomarker MALDI-TOF MS and Vitek 2™. However, 1 isolate of *S. mitis/oralis* was misidentified as *Streptococcus pneumoniae* by Vitek 2™. Studies had initially shown some difficulty in identification between these two species that was attributed to lack of extensive database [14, 22]. For *Streptococcus pyogenes* and *Streptococcus agalactiae*, biomarker MALDI-TOF MS was compared to serotyping which is conventionally used in identification in our laboratory. Biomarker MALDI-TOF MS correctly identified all to the genus and species level while the serotyping misidentified 1 to the species level.

In the identification of yeasts biomarker MALDI-TOF MS performed as well as Vitek 2™ with sensitivity of 100.0 and 95.7% (*p* = 0.312) respectively. Both *SARAMIS™ and PAPMID™* databases were unable to identify one *Candida guillermondii* and six *Candida haemulonii* isolates. This could be attributed to lack of spectra or poor representation for these species in the databases. Previous studies have shown the sensitivity of MALDI TOF MS in the identification of yeasts to range from to 82.7 to 87.2%. In the cohort studied by Lohmann et al. there was a single isolate of *Candida haemulonii* that was not identified [6]. In various studies, *Candida auris,* an emerging multidrug resistant organism, has been misidentified as *Candida haemulonii* by Vitek 2 system [23–25]. This underscores the importance of incorporation of spectra in the database for adequate discrimination between these closely related species.

A limitation of the study is the few numbers in some of the groups of microorganisms like *Streptococcus pneumoniae*, *Cryptococcus* and *Candida* species. *Salmonella typhi* was not analysed since it did not meet the shipping criteria. A larger study including these microorganisms would yield more information on the performance of biomarker based MALDI-TOF MS. We also recommend studies on the performance of this biomarker based database on direct identification from blood culture broths as recent studies have shown improved clinical utility [26].

## Conclusion

Our study has shown good performance of the biomarker based approach in a clinical laboratory setting with high sensitivities in the identification of clinically relevant microorganisms. We recommend the adoption of this approach in clinical laboratory settings to improve sensitivities and to reduce the need for molecular testing.

## Abbreviations

ACN: Acetonitrile
AKUH, N: Aga Khan University Hospital, Nairobi, Kenya
CAMPY: Campylobacter agar
CHCA: Alpha–cyano-4-hydroxycinnamic acid
CLED: Cystine Lactose Electrolyte Deficient
CoNS: Coagulase negative Staphylococci
GBA: Gentamicin Blood Agar
IBM: International Business Machines Corporation
MALDI-TOF MS: Matrix Assisted Laser Desorption Ionization-Time of Flight Mass Spectrometry
PAPMID™: Putative Assigned Protein Masses for Identification Database
rRNA: Ribosomal Ribonucleic Acid
SARAMIS™: Spectral ARchive And Microbial Identifications System
SDA: Sabouraud Dextrose agar
SOPs: Standard operating procedures
SPSS: Statistical package for the social sciences
TFA: Trifluoroacetic acid
ul: Microliter

## References

[1] Karas M, Bachmann D, Hillenkamp F (1985). Influence of the wavelength in high-irradiance ultraviolet laser desorption mass spectrometry of organic molecules. Anal Chem [Internet].

[2] Karas M, Bachmann D, Bahr U, Hillenkamp F (1987). Matrix-assisted ultraviolet laser desorption of non-volatile compounds. Int J Mass Spectrom Ion Process [Internet].

[3] Karas M, Hillenkamp F (1988). Laser desorption ionization of proteins with molecular masses exceeding 10,000 daltons. Anal Chem [Internet].

[4] Hillenkamp F, Karas M, Beavis RC, Chait BT (1991). Matrix-assisted laser desorption/ionization mass spectrometry of biopolymers. Anal Chem [Internet].

[5] Claydon MA (1996). The rapid identification of intact microorganisms using mass spectrometry. Nat Biotechnol.

[6] Lohmann C, Sabou M, Moussaoui W, Prévost G, Delarbre JM, Candolfi E (2013). Comparison between the biflex III-biotyper and the axima-saramis systems for yeast identification by matrix-assisted laser desorption ionization-time of flight mass spectrometry. J Clin Microbiol.

[7] Chean R, Kotsanas D, Francis MJ, Palombo E a, Jadhav SR, Awad MM (2014). Comparing the identification of Clostridium spp. by two Matrix-Assisted Laser Desorption Ionization-Time Of Flight (MALDI-TOF) mass spectrometry platforms to 16S rRNA PCR sequencing as a reference standard: a detailed analysis of age of culture and sample. Anaerobe [Internet].

[8] Ziegler D, Pothier JF, Ardley J, Fossou RK, Pflüger V, de Meyer S, et al. Ribosomal protein biomarkers provide root nodule bacterial identification by MALDI-TOF MS. Appl Microbiol Biotechnol [Internet]. 2015;99(13):5547–62. [cited 2017 May 24]. 10.1007/s00253-015-6515-3 25776061

[9] Suarez S, Ferroni a, Lotz a, Jolley K a, Guerin P, Leto J (2013). Ribosomal proteins as biomarkers for bacterial identification by mass spectrometry in the clinical microbiology laboratory. J Microbiol Methods.

[10] Toh BEW, Zowawi HM, Krizova L, Paterson DL, Kamolvit W, Peleg AY (2015). Differentiation of Acinetobacter genomic species 13BJ/14TU from Acinetobacter haemolyticus by use of Matrix-Assisted Laser Desorption Ionization-Time Of Flight Mass Spectrometry (MALDI-TOF MS). J Clin Microbiol [Internet].

[11] Cherkaoui A, Hibbs J, Emonet S, Tangomo M, Girard M, Francois P (2010). Comparison of two matrix-assisted laser desorption ionization-time of flight mass spectrometry methods with conventional phenotypic identification for routine identification of bacteria to the species level. J Clin Microbiol [Internet].

[12] Guo L, Ye L, Zhao Q, Ma Y, Yang J, Luo Y (2014). Comparative study of MALDI-TOF MS and VITEK 2 in bacteria identification. J Thorac Dis [Internet].

[13] Wang W, Xi H, Huang M, Wang J, Fan M, Chen Y (2014). Performance of mass spectrometric identification of bacteria and yeasts routinely isolated in a clinical microbiology laboratory using MALDI-TOF MS. J Thorac Dis [Internet].

[14] Seng P, Drancourt M, Gouriet F, La Scola B, Fournier P-E, Rolain JM (2009). Ongoing revolution in bacteriology: routine identification of bacteria by matrix-assisted laser desorption ionization time-of-flight mass spectrometry. Clin Infect Dis [Internet].

[15] Martiny D, Busson L, Wybo I, Ait El Haj R, Dediste A, Vandenberg O (2012). Comparison of the Microflex LT and Vitek MS systems for routine identification of bacteria by matrix-assisted laser desorption ionization - time of flight mass spectrometry. J Clin Microbiol.

[16] Khot PD, Fisher MA (2013). Novel approach for differentiating shigella species and *Escherichia coli* by matrix-assisted laser desorption ionization-time of flight mass spectrometry. J Clin Microbiol.

[17] Brenner FW, Villar RG, Angulo FJ, Tauxe R, Swaminathan B (2000). Salmonella nomenclature. J Clin Microbiol.

[18] Neville SA, LeCordier A, Ziochos H, Chater MJ, Gosbell IB, Maley MW (2011). Utility of matrix-assisted laser desorption ionization-time of flight mass spectrometry following introduction for routine laboratory bacterial identification. J Clin Microbiol.

[19] Lee SY, Shin JH, Park KH, Kim JH, Shin MG, Suh SP (2014). Identification, genotypic relation, and clinical features of colistin-resistant isolates of Acinetobacter genomic species 13BJ/14TU from bloodstreams of patients in a university hospital. J Clin Microbiol [Internet].

[20] Zhou C, Hu B, Zhang X, Huang S, Shan Y, Ye X (2014). The value of matrix-assisted laser desorption/ionization time-of-flight mass spectrometry in identifying clinically relevant bacteria: a comparison with automated microbiology system. J Thorac Dis [Internet].

[21] Fang H, Ohlsson AK, Ullberg M, Özenci V (2012). Evaluation of species-specific PCR, Bruker MS, VITEK MS and the VITEK 2 system for the identification of clinical Enterococcus isolates. Eur J Clin Microbiol Infect Dis.

[22] Clark AE, Kaleta EJ, Arora A, Wolk DM (2013). Matrix-assisted laser desorption ionization-time of flight mass spectrometry: a fundamental shift in the routine practice of clinical microbiology. Clin Microbiol Rev [Internet].

[23] Kathuria S, Singh PK, Sharma C, Prakash A, Masih A, Kumar A (2015). Multidrug-resistant Candida Auris misidentified as Candida haemulonii: characterization by Matrix-Assisted Laser Desorption Ionization-Time Of Flight Mass Spectrometry and DNA sequencing and its antifungal susceptibility profile variability by Vitek 2, CLSI broth microdilution, and Etest method. J Clin Microbiol [Internet].

[24] Ben-Ami R, Berman J, Novikov A, Bash E, Shachor-Meyouhas Y, Zakin S (2017). Multidrug-resistant Candida haemulonii and C. auris, Tel Aviv, Israel. Emerg Infect Dis [Internet].

[25] Okinda N, Kagotho E, Castanheira M, Njuguna A, Omuse G, Makau P, et al. Candidemia at a referral hospital in sub-Saharan Africa: emergence of Candida auris as a major pathogen. European. European Conference On Clinical Microbiology And Infectious Diseases; Barcelona, Spain. 2014.

[26] French K, Evans J, Tanner H, Gossain S, Hussain A. The clinical impact of rapid, direct MALDI-ToF identification of bacteria from positive blood cultures. [cited 2017 May 8]; Available from: http://journals.plos.org/plosone/article/file?id=10.1371/journal.pone.0169332&type=printable. 10.1371/journal.pone.0169332 PMC5201237 28036369

